# Anatomy and Physiology

**Published:** 1838-07

**Authors:** 


### PART THIRD.

### Selections from the Foreign Journals.

---

### ANATOMY AND PHYSIOLOGY.

### *Microscopical Observations on the Anatomy of the Aqueous Capsule and System of the Crystalline Lens.* By Dr. Werneck, of Salzburg.

*Aqueous Capsule.* Dr. Werneck first of all directs our attention to the development, or, as he terms it, *genesis*, of the membrane of the aqueous humour. He states that, when he examined the eye of the adult, by means of the knife and forceps, he could not convince himself of the existence of that membrane. It is only after boiling in water, and when the cornea is in a great measure reduced to glue, that the membrane of the aqueous humour can easily be separated. In the horse's eye, however, and still more readily in the eye of the lynx, especially in young animals of these genera, and also in the eye of the human fœtus, this membrane may be demonstrated without difficulty. It covers completely the posterior surface of the cornea and the anterior surface of the iris: it is closely applied to the cornea by means of short, firm, cellular membrane; but it runs loosely over the iris, descending into the depressions formed by the iridal vessels. In the fœtus, while the pupillary membrane is still entire, the aqueous capsule forms a short, compressed sac: indeed, it forms the anterior part of the pupillary membrane. There are many anatomists who teach that the aqueous capsule covers also the posterior surface of the iris, the ciliary processes, and the anterior surface of the crystalline capsule, so as to form a serous sac, lining both the anterior and the posterior aqueous chambers. Other anatomists deny the existence of this serous investment of the uvea and ciliary processes, and urge that, in that case, by dissecting the eye under water, such a membrane should be detected, and the pigmentum nigrum should not be so easily detached. Dr. Werneck describes minutely the state of the aqueous chambers, and of the pupillary membrane, during the fourth and at the beginning of the fifth month of pregnancy, towards the end of the fifth month, towards the middle of the sixth month, at the beginning of the seventh, in the course of the seventh, in the eighth and ninth months, and soon after birth. His description, however, does not differ in any important respect from that which is usually given; viz. that these cavities are formed by two shut sacs, in contact with one another at the pupil, where their apposition forms the double pupillary membrane. He describes the anterior lamina of the pupillary membrane as presenting itself in the course of the seventh month, perfectly clear and transparent, while the posterior is full of blood-vessels. The gradual attenuation of the pupillary membrane, its loss of vascularity, its rupture, and the shrinking of its fragments, have been carefully observed by Werneck: he states that, after birth, it is impossible to demonstrate the membrane of the aqueous humour, passing over the crystalline capsule. Towards the middle of the sixth month, injections through the aorta are generally very successful in filling the vessels of the eye; and indeed, without injections, the arteria centralis may be seen at that period, ramifying in a beautiful manner in the cellular substance between the posterior crystalline capsule and the vitreous body, as well as the vessels of the anterior crystalline capsule, which are partly derived from the circulus arteriosus Mascagni, partly from the ciliary processes. Werneck gives a very beautiful representation of the latter vessels from nature.

*Crystalline Capsule.* Dr. Werneck represents the crystalline capsule as a complete sac, bound down by its posterior segment to the fossula hyaloidea by means of a very short cellular membrane. Around its obtuse edge lie the retina, corpus ciliare, ciliary processes, and zonula Zinnii. The processes of the zonula Zinnii end in very delicate pointed fibres, not easily counted, which converge uniformly towards the capsule. It is here, according to Dr. W., that the membrane of the aqueous humour, after covering the anterior surface of the ciliary processes, unites with these processes of the zonula Zinnii, having loosely invested the former. The continuation of this united structure, from the points of the ciliary processes to its union with the capsule, is the peculiar structure called by Ammon *orbiculus capsulo-ciliaris*. According to Dr. W., the crystalline capsule consists properly only of one membrane; which, however, may be divided, by different manipulations, into two lamellæ. The external lamella is completely transparent, so as not, even under the microscope, either in the fœtus or in the adult, to present any characteristic structure. The blood-vessels do not appear to penetrate its texture, but to run over the surface which it turns towards the lens. No trace of lymphatics is detected in this membrane, but it appears completely clear like crystal, bringing to mind the remark of Rudolphi, that the dense smooth part of the serous membranes seems to consist of an exceedingly thin layer of cartilage, and like that is bloodless. This character of the capsule is very distinct in the lynx and beaver, but still more so in fish. This membrane, even after maceration for months in alcohol, never becomes cloudy, but retains its transparency: it does not putrefy upon being exposed to the air, but dries quickly, like any other piece of cartilage; and, being again moistened with water or other fluid, resumes its original appearance. Immediately on the internal surface of this *lamina crystallina* lies the other layer, which, in its structure as well as in its other properties, is very different. Dr. Werneck represents this lamella as seen through the microscope. In examining it, it is necessary to lay the anterior surface of the capsule, without folds, and moistened with a little water, upon a thin plate of glass, and so bring it under the compound microscope. It is formed of a loose texture, so that Dr. W. compares it to mucous membrane: it is, however, when quite recent, perfectly transparent. This lamella is exceedingly thin, measuring, after it is hardened in spirits, about the $\frac{1}{20000}$th part of a line in thickness. Under the microscope, there are observed in it many distinctly marked circular plates, the most of which measure $\frac{4}{10000}$ parts of a line. Dr. W. seems doubtful whether he should regard them as little vesicles. Between these round bodies very fine vessels form so regular a network, that one of these plates is placed within every mesh.

Although this delicate membranous layer cannot be demonstrated in an isolated state, still, by macerating it for a little time in alcohol, whereby it becomes opaque, it may be perceived with the naked eye, and scraped away in shreds from the cartilaginous lamella, which remains perfectly transparent. However much Dr. W. has laboured, and however often he has completely succeeded in injecting the fœtal eye with copal varnish, so as to show the vessels of the *lamina crystallina* as innumerable ramifications, he has never been able to discover a single red vessel in this membranous layer, although the appearance and distribution of its vessels correspond more with those of blood-vessels than of lymphatics. This delicate layer readily putrefies on maceration in water. It exists in all animals; only the little circular plates he has not been able to find, except in one species of ape, whose eyes he examined an hour after death: in most instances he saw only a structure consisting of confused scales. He regards this layer as the peculiar structure by which chiefly the liquor Morgagni is secreted, and thinks that this fluid deserves more attention than has hitherto been bestowed upon it, as upon it in part depends the life of the lens. In connexion with this layer there is still another structure, to which he gives the name of *Fächergewebe*, (honeycomb tissue,) through the medium of which the organic connexion is effected of the capsule and the lens. It is a very common opinion that the lens, like the simplest kind of hydatid, is enclosed within, but without any connexion with, its capsule. The lens, it is true, has no immediate connexion with the red-blood system, yet it enjoys no peculiar independent life. No arteries pass into the lens from the blood-vessels of the capsule, but on all sides serous vessels pass into it and nourish it.

*Honeycomb Tissue.* According to Dr. W., a peculiar honeycomb-like connexion between the capsule and the lens is to be seen with the microscope. This texture resembles in delicacy the original primitive texture, (the *Urthierstoff* of Seiller,) and consists of extremely short six-sided cells, each being about $\frac{10}{10000}$ parts of a line in thickness. This texture is most abundant behind the lens: it may be seen, however, by removing the cornea of a recent eye under water, opening the capsule by a puncture in its middle, and cautiously separating a strip of the capsule with a pair of forceps all the way to its edge, whereby the cells, which adhere more firmly to the capsule than to the surface of the lens, are torn through, and then, snipping the separated portion across, laying it on a plate of glass in such a way that the surface formerly in contact with the aqueous humour may touch the glass. So disposed, and covered with a very little water, the shred of capsule is to be brought under a microscope, magnified linearly 110 times, when the honeycomb texture will be distinctly seen, especially at the edge of the capsule. With one of Plössl's instruments, an experienced observer will see traces of the torn cells, even on the anterior surface of the lens, if it has been previously coloured with an alcoholic decoction of Brazil wood.

As the cells or texture which the honeycomb-work constitutes is exceedingly delicate, (much more so even than that which passes, in the eye of the fœtus, from the periphery of the capsule to the uvea,) it happens that, even with the most careful manipulation, the greatest part of the cells are distorted, and their symmetrical appearance destroyed. The fine substance by which these six-sided cells are formed is very transparent, but, like the inner lamella of the capsule, becomes cloudy when put into strong alcohol; never so much so, however, as the membrane of the hyaloid cells.

In the space of one of these cells,. four or five of the circular plates of the internal lamella of the capsule are observed. The depth of these cells Dr. W. has not ascertained: judging, however, from the distance between the capsule and the lens, they cannot surpass $\frac{16}{10000}$ parts of a line. Through these cells, which communicate with one another, the humor Morgagni flows in upon the lens. Although some doubt that this humour exists during life in any appreciable quantity, or believe that it appears only after death, from imbibition or transudation, the oculist, who has seen what is styled *cataracta Morgagniana* even only once, will not hesitate about the existence of this fluid. Dr. W. formerly thought that, in the living and healthy state, the liquor Morgagni formed merely a vapour; but he is now convinced that it is an actual fluid. It will not surprise those who know from experience the rapid power of absorption of the vessels of the lens, and reflect that, in the act of death, when the *turgor vitalis* fails, the capillary system, in most cases, dies sooner than the heart,—and so all the secretions cease while absorption goes on, that, when the eyes of animals are examined soon after death, the humor Morgagni is found in so small a quantity, or not at all.

*Lens.* Whoever examines the lens in man and other animals, in its different stages of development, as well as at different periods of life, in its healthy as well as in its diseased states, and compares it with other organic structures, will be convinced that it is composed in a great measure of fibrous bundles, which are regularly laid upon each other, so as to form thin leaf-like layers, like the coats of an onion. Each concentric leaf, or shell, presents two different organic structures, more especially demonstrable in the fœtal lens, which has lain in some coloured fluid containing tannin.

1. *Membranous Part of the Lens.* At each pole of the lens we meet with a very fine, membranous, porous, completely transparent structure. At the anterior pole this structure forms three slender lines, meeting at equal angles, towards which lines the fibres, afterwards to be described, run, and to which they are connected: so that the anterior surface of every lamella is divided into three segments. In these three lines the fibres are lost, as the muscular fibres in a tendinous expansion; but, at the points of the three converging lines, the fibres run in a vorticose manner, something like the fibres of a sphincter. On the posterior surface of the lens, this membranous structure is commonly formed of four lines, sometimes only of three. In a man of ninety-six, Dr. W. found only a small disc, not quite round, whence the fibres radiated towards the periphery of the lens. On this surface, as on the anterior, the fibres form a kind of vortex at the point of each of the three or four lines; while, towards the centre, where the lines meet, the fibres run nearly in a straight direction. It is this delicate texture which becomes torn in living animals when the solar light, or phosphorus light,* is directed upon the crystalline by means of a lens or concave speculum. When this is done, the crystalline flies into three or four pieces. This fine membrane passes from the poles inwards, and spreads out from the axis of the lens between the laminæ. On macerating the lens in water, this fine texture, which forms the poles and the spaces between the lamellæ, remains behind, so that it may be examined in water, or, which is better, in alcohol.

The three converging lines on the anterior surface of the lens are not opposite to the three on the posterior surface. We believe that, on the anterior surface, one of the three lines runs horizontally towards the nasal edge of the lens, while the other two lines diverge from it towards the temple; while, on the posterior surface, one of the three lines runs towards the temple, the other two diverging towards the nasal edge of the lens. This, however, is contrary to the statement of Dr. W.: he says that, on the anterior surface, one of the three converging lines is usually directed upwards, and the two others towards the sides. Dr. W. states that the three lines on the anterior surface of the lens are constant, but that he has met with slight deviations on the posterior surface in domestic animals. In the rodentia, the anterior surface of the lens presents a single transverse line, and the posterior a single vertical line; the same also in fish; so that the lens in these animals divides into four segments. In birds and the amphibia, there is no trace of segments: only in the middle, where the fibres meet, there is a small round structure, an actual membranous pole. In the adult lens, the segments are completely united, so that the lens can be separated only by mechanical or chemical means. This union of the segments is accomplished by means of the exceedingly fine cellular membrane, forming what Dr. W. calls the membranous part of the lens. It is only in the eye of the fœtus that this cellular connexion can be demonstrated clearly and completely. At this period, also, the three lines on the anterior and posterior surfaces of the lens are much broader, and vanish gradually in proportion as the roundish form of the crystalline changes to the lenticular.

2. *Fibrous Structure of Lens.* This structure forms the greatest part of the laminæ of the lens. Each fasciculus of fibres measures, in the superficial laminæ, $\frac{3-4}{10000}$ parts of a line: each such fasciculus contains four or five fibres. In the deeper lamellæ, the fasciculi, as well as the fibres themselves, appear to be thinner and more delicate. These fasciculi are quite perceptible when the lens is submitted to the microscope, enclosed in its capsule. The fasciculi can be torn into fibres, in the direction of their length, which cannot be done with lymphatic vessels. These fibres and the lamellar structure of the lens are not produced by hot water, alcohol, mineral acids, or mineral solutions; no more than by these means we can change the structure of the brain or of muscular fibres. Why (Dr. W. asks,) shall we not regard these fibres of the lens as similar to muscular fibres? Some may answer, because, when they are separated, or when they are rubbed, as Berzelius directs, they almost entirely dissolve in cold water; which does not happen to fleshy fibres. Dr. W. says, that not to mention that, through division and trituration, the whole organization of so delicate a structure is destroyed, he shall only state, by way of answer, the fact that the lens presents, in this respect, a similar condition with the muscles of many water-insects, and especially the rotatoria; and that all the organs in the eye present quite a peculiar structure,

* By phosphorus light, we suppose Dr. W. means the light obtained by burning phosphorus in oxygen.

especially those which possess no red vessels. Also, in a morbid state of the lens, especially in arthritic cataract, we distinctly observe the fibrous concentric structure of the lens.

Chemistry affords us very little information on the peculiar composition of the lens. Fifty per cent. of it is water; the rest chiefly a peculiar matter which seems to hold a middle place between albumen and osmazome.

Of the spaces between the lamellæ, it is easy to convince one's self by allowing the lens to lie for several days in distilled vinegar, coloured with carmine; as, by the absorption of this, these spaces become filled with the colouring matter. In these spaces the Morgagnian fluid circulates, and every lamina is enclosed the one by the other; each capsule, and with it the membranous part, becoming smaller and smaller, and more and more delicate, till we come to what is called the *nucleus* of the lens.

*Development of the Lens.* Notwithstanding numerous researches in the embryos of quadrupeds, birds, and fish, Werneck has not been able to determine whether the eye is projected from the middle ventricles of the brain, or formed, originally and independently, out of the primitive animal tissue. He is certain that the lens, however, lies close to the fibres of the optic nerve, and is developed quite independently of the other parts, and without any blood-vessels being discovered in it. Already, by the end of the second day, in the chick, a trace of lens is seen, like a round elementary corpuscle. In the third day, the capsule is distinctly perceived, like a completely round vesicle. It is entirely shut and perfectly transparent; its contents are somewhat pappy, and consist, as seen under the microscope, of opaque, milky-coloured, partly round, partly oval grains, enclosed within a gelatinous material. These Dr. W. regards as primitive, granular substance. In the fourth day, the retina, forming already a completely fissured membrane, covered with blackish grains at the sides, and traces of the uncoloured edge of the iris, also fissured, appearing, there forms within the capsule of the lens, at every point of its internal surface, a peculiar cellular texture, which is the connexion of the lens with its capsule. In the beginning of the fifth day, the zonula Zinnii being already formed, the fibres of the lens begin to be visible towards its edge. The embryonic grains run in wreaths from the edge towards the centre, and, from the running together of these grains, the fibres of the lens are formed. This change does not take place piecemeal or by laminæ, but in the whole of the original mass of the lens the fibres are formed. There is no appearance of lobes or segments. At the end of the fifth day, the fibres, running from the periphery to the centre, are still more distinct; and, as the formation of the fibres proceeds, the lens from its periphery becomes clear. In the seventh day, when the ciliary processes show themselves, the granular, original substance retires still more, and the formation of fibres advances. By the tenth or eleventh day, the clearing of the periphery is far advanced, and the grains are only at the middle, but already arranged in the form of fibres. By the thirteenth day, the circumference of the lens is completely clear; its middle only is somewhat cloudy, but the grains are no longer visible.

The lens is formed exactly after the same manner in the amphibia; so also in fish, only that in them the development goes on in four segments. On the sixth day in rabbits, and on the fourteenth day in the calf, the formation of fibres out of the embryonic material is already evident within the completely round capsule; but the formation of the fibres goes on in these animals, as well as in the hare, Guinea-pig, &c., at once in four groups; in cattle, sheep, swine, and man, in six. Even here, however, the formation of fibres out of the original material does not take place by layers from the centre to the periphery, but the reverse: the fibres form in the whole extent of their groups, from the periphery towards the centre. In the calf already at the beginning of the second month, and in man in the course of the first fourteen days, the first trace of the formation of the lens is visible. By the end of the third week, the formation of the crystalline fibres is considerably advanced; the lens at its circumference is already transparent, and the segments are already half united. Werneck believes that, when the fibres have formed small segments of a sphere, such segments unite two and two together, and become so joined by the cellular substance already mentioned, that they form, commonly in cattle, swine, and man, a third segment of the lens; but in hares, rabbits, and fish the half. The union of the segments immediately follows. The traces of the union of the segments form the lines of demarcation which are well known.

In the calf, the cloudy appearance of the lens vanishes in the eighth week; in man, sometimes in the eleventh, and sometimes not till the fourteenth week. In pheasants, leverets, and partridges, Dr. W. repeatedly saw the cloudiness in the middle of the lens gradually vanish after birth. In the sixth month, the middle of the lens begins to turn firmer; and, in the eighth, the kernel is to be found, though still delicate, like all the textures of the fœtus.

Dr. W. represents the individual crystalline fibres in man, the mammalia generally, and in birds, as having the form of six-sided, elongated prisms, which in old animals become stiff externally, and are thus harder than in their interior. These fibres, in a separated lamina, are of unequal length, breadth, and thickness. At their extremities they are slenderer, and terminate in blunt points; they are broadest in their middle, where they form the periphery of the lens. The longest fibres occupy the middle of each lamina; at the sides of the laminæ, where, when the laminæ are joined together, the vortices are formed, the fibres are shortest. In the middle of the laminæ they are straight; at the sides they are bent.. In the mammalia, some fibres are observed not to run the whole length of the laminæ to which they belong; and sometimes, in cattle, they run in a waving manner.

In birds and the amphibia, the course of the fibres is more regular, and their extremities become more rapidly pointed. In man, they are about the most slender, and, on maceration in water, they dissolve into fine threads. The deepest fibres are the most slender.

In fish and the amphibia, the fibres have the form of flat, little bands, the edges of which are variously indented according to the species; the indentations reciprocally receiving and being received, like the sutures of the skull. In man, the mammalia, and birds, the connexion of the fibres is accomplished in such a way that each fibre, with its prismatic surfaces, is received between two other fibres.

The lens consists of layers of different densities; and it is easy to distinguish the external, softer, and more glutinous laminæ from the internal, which are more consistent. On the various densities of its laminæ, their various curvatures, and the various positions of its kernel, depend the various refractive power of the lens. Treviranus distinguishes three different consistencies in a section of the lens, which gradually run into one another. In birds, the kernel lies always in the centre; not so in all the mammalia, though it is the case in man. If we macerate the lens of a newborn mammiferous animal, it is found that the central part is always exceedingly firm; but this does not consist of crystalline substance, but of cellular membrane, what Dr. W. calls the *membranous part* of the lens, which remains undissolved. Dr. Werneck contends that, in the lens of the embryo, there is no liquor Morgagni; and that, of course, the crystalline substance cannot be regarded, as some have been inclined to do, as a deposition from, or crystallization of, the Morgagnian fluid. He considers it an unlikely supposition that the honey-comb tissue, which serves as connexion between the capsule and the lens, can deposit the substance of the fibres as we find them, in various animals; of prismatic form, and with their edges sometimes smooth, sometimes denticulated, and arrange them so that each fibre is laid between two neighbouring fibres.

Dr. Werneck's communications are accompanied by no fewer than forty-six figures, illustrative of the structure of the capsule and lens in man and other animals.

The following curious experiments on the eye are mentioned by him in a note: In a cataractous rock-shrite and an English hound, Dr. W. disruptured the cataractous lens by the use of a strong burning-glass, and had the pleasure to find that it gradually dissolved, and at length was totally removed. The retina was not at all injuriously affected by this experiment, nor did inflammation arise in consequence of it. He asks, whether this same means might not be used in the human eye affected with cataract, as well as on that of the lower animals, when the capsule is not much thickened, or the cataract purely lenticular. If the experimenter is careful not to bring the focus of the lens upon the cornea, but only permits it to fall for a moment on the surface of the capsule, there will be no danger.

As he wished to convince himself of the cause of this disruption of the lens, which could not consist in any action of the muscles of the eye alone, he opened the cornea in a number of animals, removed the iris, and then convinced himself, during the use of electricity, and while viewing the eye through a powerful glass, that the disruption of the lens was chiefly brought about by strong spasmodic contractions of the zonula Zinnii. In this very interesting experiment, he recognized the truth of Camper's opinion that the fibres of the zonula Zinnii are muscular, and thinks that Döllinger's explanation is not to be rejected, when it is evident that, through the contraction of this ring, the lens is pushed forward, and brought nearer to the uvea. He thinks this motion of the lens, taken in connexion with its lamellar structure, and the action of the muscles of the eye, ensures distinctness of vision at different distances.

*Ammon's Zeitschrift.* B. iv. H. 1, B. v. H. 4. 1837.

---

*On Reflex Motions.* By Dr. A.W. VOLKMANN, Professor of Physiology at Dorpat.

[IN the following article we have given all that is of any importance in Dr. Volkmann's paper. In several of his remarks and conclusions in the latter part of it, (less novel than the author seems to imagine,) we do not concur; but we have been unwilling to omit anything that bears on a subject at this moment so livelily discussed, and possessing such intrinsic interest.]

1. *Phenomena observed in Frogs after Decapitation.* On dividing the spinal column of a frog immediately below the skull, violent muscular motions are induced, which, however, are not of long continuance. Sometimes the posterior extremities are adducted to such a degree that the body makes a complete somerset; at other times the effect is entirely opposite, the extremities being violently extended. Dr. Hall has asserted that the decapitated animal, after the first disturbance produced by the operation has subsided, remains quietly in the position which it then assumed till the extinction of life, if no external excitant be applied. This is a point of great physiological importance, but Dr. Volkmann found the assertion to be erroneous. This physiologist has repeatedly observed decapitated frogs call the posterior extremities into action, without any external cause being applied, for the purpose, apparently, of assuming a more comfortable position. It is generally remarked that, after the head has been separated from the body, and the convulsive motions have subsided, a period of quietude succeeds, the result apparently of exhaustion. In this period, generally a few minutes after decapitation, there is an interval in which the body is little irritable, and, although at a later period the slightest touch produces reflex motions, it may then be handled without producing these movements. If, when in this condition, the body be extended on a hard surface, it will retain the position in which it has been put for five or ten minutes, till suddenly, and without the application of any external stimulus, it will draw up the thighs, and change its recumbent to the sitting position; in which, however, it will remain, in all probability, till the extinction of life.

2. *The simplest Phenomena of Reflex Motion.* On irritating a decapitated frog by pricking, pinching, burning, or even merely by touching it with the finger, muscular motions are induced, which correspond exactly with those of voluntary motion, and are not merely sudden convulsive twitches, such as ensue on irritation of a muscle. These phenomena continue in lively frogs, salamanders, and water salamanders, always for several, often for many, hours after decapitation; but cease immediately on the destruction or excision of the spinal cord. If any of these amphibia be divided into several parts, each part which contains a portion of the spinal cord will exhibit reflex motions, which cease in each part with the destruction of the corresponding portion of the spinal marrow.

3. *Reflex Motions, with longitudinal Section of the Spinal Cord.* The upper part of the spinal cord of a decapitated frog was laid open, and the cord divided in a longitudinal direction from its commencement, as far as the fourth spinal nerve. Uninterrupted convulsive twitchings of the anterior extremities, and slight convulsions of the posterior extremities, were the consequence; and, when they had subsided, the animal assumed the sitting position. On touching one of the anterior extremities, it was withdrawn; and, on pinching it severely, not only was the irritated foot withdrawn, but both the posterior extremities were violently moved. On reapplying the irritation, the same phenomena were observed; but no irritation of one anterior extremity produced motion in the other. On irritating the posterior extremity, reflex motions were induced, not only in the opposite extremity and in the anterior extremity of the same side, but also, to all appearance, in the anterior extremity of the opposite side; for, on pinching the left posterior extremity, the animal threw itself over on its left side, which could only be effected by extension of the right arm: convulsive motions were at the same time observed in the muscles of the right shoulder, which derive their nerves from that part of the cord which had been longitudinally divided.

The lower portion of the vertebral canal of a decapitated frog was now laid open, and that part of the spinal cord which supplies the posterior extremities with nerves divided longitudinally. Irritation of the anterior extremities was followed by motion of all four; but, on irritating one of the posterior extremities, distinct reflex motions were perceptible only in the anterior extremity of the corresponding side: in the anterior extremity of the opposite side they were doubtful, and in the opposite posterior extremity not a trace of motion could be perceived. In another frog, the spinal cord was divided longitudinally from its commencement, as far as the last lumbar nerve; but in this case the reflex motions were very indistinct; a result apparently dependent upon the great organic destruction. Irritation of the anterior extremities produced no movements in the posterior; irritation of a posterior foot produced motion in the muscles of the corresponding thigh and side of the abdomen, and, when very severe, even in the musculus cucullaris of the same side; but there was never the slightest movement in the muscles of the opposite side of the body, although the spinal cord below the sciatic nerve remained uninjured.

In another instance, the whole of the vertebral canal, excepting the fourth and fifth vertebræ, was laid open, and the cord divided longitudinally, except where it was covered by these two vertebræ; thus leaving a small portion of the cord, supplying the middle part of the abdominal region with nerves, uninjured. Irritation of an anterior extremity produced reflex motions in it alone; but irritation of a posterior extremity was followed by motion of all four.

In drawing conclusions, proof can be deduced from those cases only in which motion occurred, not from those in which it failed; for it is evident that reflex motions can take place only when their organic conditions are fulfilled; whereas, they must often fail on account of the great destruction produced by the knife. With these considerations in view, Dr. Volkmann thought himself justified in assuming that longitudinal division of the spinal cord does not prevent the extension of reflex motions over all the muscles of both sides of the body, so long as any part of the proper cord remains united in the mesial line. The proper cord he limits to that part which gives origin to the first ten spinal nerves; the part posterior to this does not seem to be capable of reflecting stimuli from one half of the body to the other.

4. *The Reflex Motions partake of the character of Adaptation to a determinate end.* The motions which ensue on irritating a decapitated frog not only sympathise in so far as those muscles which are associated together in life are thereby called into simultaneous action; but that action is of a kind adapted to the attainment of an object, for the nature of the movement varies according to the peculiar nature of the stimulus. A decapitated tortoise, when irritated, conceals itself beneath its shell; and a decapitated frog, on being irritated, comports itself according to the nature and degree of the irritation. The fore-feet of a frog were irritated, and it withdrew them: they were further irritated, and it withdrew them further: they were still further irritated, and it then drew them in below the belly, and changed its sitting position into the recumbent. If the posterior extremity of a decapitated frog be violently irritated whilst it is in the sitting position, it will bound forwards: if it be roughly seized in the thoracic region, it will plant its fore-feet upon the hand which holds it, and try to free itself; and, when the skin of the abdomen or back is seized with the forceps, it is by no means uncommon for the mutilated animal to scratch the part with the posterior extremity of the corresponding side.

[From these experiments Dr. Volkmann concludes "that the decapitated animal is aware of the action of the stimulus, and chooses, amongst a variety of means, those which are best calculated to free itself from the annoyance;" but we cannot see that this inference is warranted by the facts. It involves the supposition that not only sensibility but the power of willing remains in the spinal cord after decapitation; and not simply in the spinal cord, but in each segment of it, when separate from the rest. On this hypothesis, we might separate, not plants and polypes only, but human beings, into as many distinct individuals as each possesses pairs of spinal nerves; since we cannot conceive any higher qualification for a separate individuality than the power of feeling and of willing. The mere adaptation of particular movements to designed ends cannot be regarded as a reason for supposing that these movements are voluntary, or even dependent upon sensation. The whole organized fabric is made up of a number of such adaptations. The motions of the heart, alimentary canal, &c. are instances of their simplest forms. The motion of the pupil is a case more directly to the purpose, being a reflex action evidently adapted to a particular purpose,—the conservation of the eye in the state best adapted for the visual function; yet every one knows this to be independent of volition, and pathology shows that it may be performed without sensation.]

5. *The Extent of the Reflex Motions is chiefly dependent upon the amount of Irritation and the degree of Excitability.* When one of the amphibia, recently decapitated, is gently irritated, the motions are confined to the parts in the immediate vicinity of the irritated spot: it happens sometimes, for instance, that tickling of a toe will produce movements which are confined to the irritated foot alone; but, if a greater degree of irritation be employed, the whole limb is thrown into motion; and, if a still greater irritation be employed, the movements extend over the whole muscular system. In the same manner, the extent of the reflex motions depends upon the degree of excitability. Some time after decapitation, irritation of a limb is insufficient to produce motion in any part of the body, except in the irritated limb itself, owing to the diminution of excitability.

6. *In the Reflex Functions the posterior Roots of the Spinal Nerves are exclusively Excitors, the Anterior Roots exclusively Reflectors.* By excitor nerves are meant those which conduct the stimulus which affects a peripheral organ to the spinal marrow; and, by reflectors, such as transmit the stimulus from the centre outwards to the muscles. After division of the three posterior roots of the sciatic plexus of a decapitated frog, the most violent irritation of the limb to which these nerves are distributed is insufficient to produce reflex motions; although, as it is easy to prove, the operation destroyed neither the reflecting power of the spinal cord nor the muscular irritability of the extremity; for, on irritating one of the anterior feet, reflex motions in all four extremities ensue. Hence it appears that, if division of the posterior roots of the sciatic plexus deprives the nerve of the power of producing reflex motions, it is because the posterior roots alone are capable of transmitting a peripheral stimulus to the spinal cord.

When, on the contrary, the anterior roots of the sciatic plexus are divided, irritation of the corresponding extremity produces reflex motions in the other three extremities, but none in itself; whilst irritation of one of the uninjured extremities produces motion in all the muscles of the body, with the exception of those of the extremity the nerves of which had been divided. It is, then, apparent that the posterior roots of the spinal nerves are incapable of transmitting a stimulus from the spinal cord downwards to the muscles.

These experiments were in imitation of those of Prof. Müller, (*Physiologie*, Band i. s. 703;) but, as this physiologist experimented upon undecapitated animals, some doubts might have been thrown upon the conclusion drawn from his experiments, as the disturbing influence of the will was not sufficiently attended to. His experiments might be said to prove incontrovertibly that the sentient filaments are the exclusive means of transmission inwardly, and the motor fibres those of transmission outwardly; but they left the point undecided whether, on the one hand the sentient and excitor filaments, and on the other the voluntary-motor and reflector fibres are identical.

7. *The Efficacy of the Stimuli which produce the Reflex Motions is modified or exalted by the extent of the Surface.* In a recently decapitated frog, the slightest touch is sufficient to produce reflex motions, although the epidermis prevents the direct action of the stimulus upon the nerves; but when, on the other hand, a nerve is exposed, it requires irritation of considerable energy to produce these movements. The great excitability of the skin is peculiarly manifest in frogs which have been brought under the influence of opium and then decapitated; tickling with a feather being in such cases sometimes sufficient to produce general convulsions, although, when a portion of the integuments has been removed, pricking and pinching of the subjacent muscles is insufficient to produce reflex motions. But, even in frogs which have not been affected with opium, the skin is possessed of a greater degree of excitability than seems to belong to the trunks of the nerves which supply it with sensation. A large flap of integument covering the whole dorsal region was separated, by an oval incision, from communication with the rest of the integuments, and was thus left in union with the body solely by some bands of cellular tissue and the cutaneous blood-vessels and nerves. Irritation of this flap with the forceps produced distinct reflex motions. It was then raised at one end, and its adhesions to the body divided. During this process, very indistinct reflex motions were only twice observed in dividing some nervous twigs, but at no other time. This experiment was repeated by isolating a large flap on the abdominal surfaces: irritation of it produced not only lively reflex motions, but also motions of adaptation; for, on pinching it with the forceps, the animal placed its fore-foot upon the irritated spot; but no reflex motions ensued on separating the flap cautiously from the body with the scissors. In dividing larger nervous trunks, such as the posterior roots of the sciatic plexus, reflex motions are occasionally observed; but they never partake of the character of adaptation. Had the animals which formed the subjects of experiment not been previously decapitated, it would very probably have been concluded that irritation of the skin produces perception of the stimulus, and, consequently, rational reaction; and that irritation of the nervous trunks excited the spinal cord only, producing organic, but no mental reaction.

8. *Irritation of the Sympathetic Nerve excites extensive Reflex Motions.* The thoracic and abdominal cavities of a frog were laid open, and, after the animal had subsided into a state of repose, those parts which derive their nerves from the sympathetic were irritated. It was thus ascertained: 1, that the reflex motions which are produced in frogs by the irritation of the sympathetic, although not so striking as in cases of cutaneous irritation, are still very decided, extending, in favorable cases, over the muscles of the abdomen, back, and four extremities; 2, that irritation of the bladder, of the large vessels in the vicinity of the liver, and of the oviducts during the period of sexual intercourse, produced the most lively reflex motions, whilst no reaction was observed to follow irritation of the heart, lungs, or liver; 3, that no reflex motions followed the irritation of the peripheral extremities of the sympathetic in the mucous membrane of the intestines, whilst they were exceedingly lively when a small nervous branch of the omentum was seized with the forceps; 4, that movements of adaptation were not observed to follow irritation of the sympathetic, although the reflex motions produced were different from the mere convulsions of muscular irritability, as was evident from their continuing after the stimulus was withdrawn, and from the requisite associated action of the muscles as flexors and extensors being induced; 5, that irritation of the intestinal canal by pinching produces contraction of the intestine, which extends upwards and downwards over a greater or smaller space; 6, that, when the spinal cord is destroyed, irritation of the intestine produces mere local contraction, by which it appears that the sympathetic ganglions have no reflecting power; and, lastly, that it does not appear that motions are produced more readily in those muscles adjacent to the irritated sympathetic branches than in those which are more remote.

[It may be reasonably questioned whether some filaments of the spinal nerves may not be the source of these reflex actions: at any rate, we know that the parts here mentioned as originating them have some connexion with the cerebro-spinal system, from the sensations of pain or distress to which these diseased states give rise. The effect upon the intestines mentioned in 5 and 6, as resulting from destruction of the spinal cord, is only an instance of what has long been known of the connexion between any violent impression on the nervous centres and the diminution or loss of general muscular irritability.]

Dr. Volkmann endeavours to show, in the following paragraphs, in how far the above experiments correspond with the prevailing doctrines of the nervous system:

*The Transmission of the Nervous Action, from the Surface to the Centre, and from thence back to the Surface, is not subject to the same law as its transmission along the Nerves, as it does not follow the course of isolated fibres.* Recent investigations have proved that the stimuli of sensation, as well as those of motion, pass only along the fibres to which they were originally communicated, and do not pass into others; and, hence, that each elementary nervous filament may be considered as isolated: but the above experiments prove that the filaments of the spinal cord are possessed of no such isolating power, but that, on the contrary, the nervous action may pass from one fibre of the medulla to another. On pricking the web of the foot of a recently decapitated frog with a fine needle, reflex motions ensue in all the muscles of the body. The point of the needle cannot have irritated above one or two nervous filaments; and it would be necessary, in order to explain the consequent phenomena upon the known laws of nervous transmission, to assume that the irritated fibres stand in direct communication with at least one motor fibre of every muscle in the body. But this would still be insufficient; for Prof. Müller has shown that partial irritation of a motor nerve produces only partial motion of the muscles; and as, in the reflex motions, the muscles are not partially but wholly thrown into action, the irritated fibres would require to communicate directly, not only with one, but with many nervous fibres of such muscle. As each fibre which transmits the irritation to the medulla can produce general reflex motions, it would naturally be supposed that the spinal cord must consist of innumerable nervous anastomoses and interlacements; a structure, however, which examination does not confirm.*

It is certain, therefore, that the elementary fibres of the nerves exert an isolating power over the nervous action; but it is equally certain that the fibres of the

* Müller decidedly refuses to admit the anastomosis of the medullary fibres, and, as Treviranus, in his Microscopical Investigations of the Nervous Substance, takes no notice of them, it may be supposed that he did not observe them. Ehrenberg, in innumerable investigations of the structure of the spinal cord noticed an anastomosis of the filaments only four times; but E. H. Weber has lately maintained that he has frequently seen the medullary fibres giving off branches to anastomose with others. Dr. Volkmann, since writing the above, was invited by Dr. Weber to attend a microscopical investigation of the structure of the brain of the pigeon, and was shown several preparations by this physiologist, which were said to exhibit the nervous anastomosis; but in three instances only was the ramification undoubted: in by far the greater number, the appearance was owing simply to the crossing of fibres, without any communication.

spinal cord are capable, under certain circumstances, of transmitting their action to each other. On irritating the crural nerve of an amputated posterior extremity of a frog in the region of the knee, all the muscles situated below the irritated point are thrown into convulsions; but none which are supplied with nerves arising above the irritated point are affected, because their nerves do not stand in union by anastomosis with the irritated point, and because there is no transmission of the nervous action from one fibre to another in the nervous cord, and, consequently, there can be no transmission of motor excitement in a centripetal direction. But, on irritating the crural nerves of a frog which has been deprived of its brain, but which retains the spinal marrow, not only are those muscles which are situated below the irritated point thrown into convulsions, but likewise those which are situated higher; a phenomenon which can be explained only on the supposition that there is a crossing, as it were, of the nervous action from fibre to fibre of the medulla.

There is no anastomosis of nervous fibres in the brain, and yet a sensation proceeding from a single sentient fibre develops an action which, by means of the will or imagination, is transmitted to innumerable motor fibres: for instance, when an individual lost in thought is suddenly pricked with a pin, the sensation causes him to jump up, and thus call into action almost all the muscles of the body. It remains to be determined why, in reflex motions, the nervous actions cross from one fibre of the medulla to another; whilst, in other cases, its fibres form conductors as isolated as those of the nerves. It deserves to be borne in mind that a stimulus produces reflex motions in decapitated amphibia, which, previous to decapitation, had no such effect; and hence it would appear that the brain contains the cause which prevents the nervous action from crossing from filament to filament in non-mutilated animals.

On consideration that all reflex motions in the body are involuntary, and in general the result of stimuli, of the operation of which we are unconscious; and that, during sleep, motions are induced by touch, which, in the waking state, either would not have taken place or would have been performed through the influence of the will; it is impossible to avoid the conclusion that the transmission of the nervous influence from filament to filament is favoured by deficient mental energy, and is, on the contrary, in certain cases, prevented by an exertion of mental power.

The will prevents reflex motions, because, of two opposing forces, the weaker must yield, and the power of the will is often sufficient to keep muscular motions in control. It is not asserted, however, that decapitation favours the production of reflex motions solely by the removal of the mental influence: on the contrary, it appears probable that there are other, but unknown, causes engaged; for the inclination of the nervous action to cross from filament to filament is so great in decapitated amphibia, that it would require a very uncommon degree of mental influence to control them, on the assumption that it forms the only controlling power.

When the degree of irritation is small, the nearest muscles only exhibit reflex motions; but, when the degree of irritation is increased, the more distant muscles likewise are called into action. Hence, it may be inferred that the transmission of the nervous action from one nervous filament to another is favoured by an increase of the degree of irritation, and that contiguous nerves communicate their nervous action easier to each other than to nerves lying more remote. This second axiom is true only in a limited extent; for, when the hind-foot of a decapitated frog is gently irritated, the motions induced are confined to the foot alone; but, when the irritation is increased, they extend likewise to the muscles of the thigh, but without implicating the muscles of the other posterior extremity. But the feet derive their nerves from the second and third roots of the sciatic plexus, whilst the muscles of the thigh are supplied from the first root. The first and third roots, however, are separated by a greater space from each other than are the third roots of the right and left extremities; and, consequently, the transmission of the nervous action appears to be easier in a longitudinal than in a transverse direction; assuming it as granted that the apparent root is the real root of the nerve.

10. *Experience has not yet sufficiently proved that all Reflex Motions of decapitated animals, and particularly of Amphibia, are independent of the cooperation of the mind; namely, of Sensation and Volition.* Dr. Marshall Hall has excluded the cooperation of the mind in every case, chiefly because decapitated animals, when they have once become quiet, remain immoveable in their assumed position, if not irritated. The incorrectness of this assertion, as far as regards tangible irritation, has already been shown; but it cannot be denied that the influence of the air upon the raw surface of the wound might be sufficient to produce reflex motions, although the admission of such stimuli would rest upon mere hypothesis. His reason for supposing that sensation is not active in producing the reflex motions are unsatisfactory: he calls attention to the circumstance that they not unfrequently cease when the body is in a position which must prove highly uncomfortable, or even painful to the animal, if it retain sensation: they ceased, for instance, in a serpent whilst the tail was hanging over the sharp edge of a table, and were not renewed by pricking it or burning it with the flame of a candle: but their non-occurrence here was owing to the exhaustion of the excitability; and this is obvious, because pricking and burning in general produce reflex motions in decapitated animals. On the other hand, those motions which we have termed the motions of adaptation speak strongly in favour of the cooperation of the mind; although it must be admitted that there are many vital functions which partake of the character of adaptation, that are altogether independent of the will.

11. *Dr. Marshall Hall's proposed Classification of the Nerves.* It has already been stated that Dr. Hall admits two kinds of centripetal nervous fibres, the sentient and the excitor; and two of centrifugal fibres, those of voluntary and involuntary motion. The former two constitute the cerebral, or sentient and voluntary system; the latter two, the spinal or excito-motory system. The reflex motions belong exclusively to the true spinal nerves; that is, to nerves of which the fibres are incapable *per se* of transmitting both sensation and volition: but, if this view be adopted, it excludes from the class of reflex motions a number of phenomena which appear to belong to them. Indeed, it may be said that every voluntary motion is reflected, as being the result of a mental act called into existence through the medium of the impressions of the senses; the received stimulus being converted by the brain into one of volition. The motions of sneezing and the contraction of the pupil cannot be separated without violence from the reflex motions, and yet they cannot be said to belong exclusively to the spinal system. It is also doubtful whether the reflex functions, in a more limited sense, are confined to the excito-motor nerves. The point of the finest needle cannot penetrate the skin without producing pain; and hence it must be inferred that each minute point is endowed with sentient fibres. But the prick with the finest needle causes reflex motions in the decapitated frog; and, if these motions be dependent solely upon the true spinal filaments, we shall be forced to admit that each minute point of integument contains two specifically different nervous fibres.

Dr. Volkmann views Dr. Hall's classification of the individual nerves as highly objectionable, scarcely one nerve being assigned a position in which it could not be assailed. The nerves of the senses have, for instance, been classed under the sentient and voluntary system, but they are equally excitors, as is proved by irritation of the retina producing contraction of the iris and closing of the eyelids. The positions assigned to the pneumo-gastric, the trochlearis, and abducens may likewise be questioned, and Dr. Volkmann objects to the ganglionic system being separated from the other nerves, without reference to the circumstance that this system of nerves includes in its composition both sentient and excito-motory filaments.

12. *On the Participation of the Nerves in Sensation.* This paragraph contains some speculative ideas on the question, whether the nerves, in conducting impressions to the brain, act merely as passive conductors, as wire in conducting galvanism; or whether they effect a change in the nature of the impression before it reaches the sensorium.

*Müller's Archiv.* 1838. Heft i.

---

*Anatomical Observations and Remarks.* By Dr. KRAUSE, of Hanover.

I. *On the Capillary Vessels.—The Thymus Gland.—The Intestinal Glands.*

1. The *capillary vessels* in many parts of the body, examined by the author, have presented a diameter considerably less than that of the smallest globule of the blood. The following is the measurement of the diameter of the most delicate capillary vessels in various parts:

| | | |
|---|---|---|
| In the retina . . . | $\frac{1}{540}$ | of a line. |
| choroid . . . | $\frac{1}{801}$ | — |
| pulmonary cells . . | $\frac{1}{801}$ $\frac{1}{898}$ | — |
| intestinal follicles . . | $\frac{1}{555}$ | — |
| muscular integument of small intestine, | $\frac{1}{740}$ | — |
| the tibialis anticus muscle . | $\frac{1}{1110}$ | — |

In proportion to the capillary vessels of the ordinary diameter, (i. e. $\frac{1}{200}$ to $\frac{1}{300}$ of a line,) these very delicate ones are always fewer in number, and are generally placed intermediately between two larger branches. Krause has never found that the larger quantity of any capillary tissue was formed by these extremely delicate vessels. The injections employed were vermilion and size, or the successive injection of a solution of neutral chromate of potass, and acetate of lead, with some mucilage of gum arabic: the granules of chrome yellow thus formed are of a diameter of from $\frac{1}{800}$ to $\frac{1}{1200}$ of a line. The granules which remain after human blood has been macerated for two days in distilled water, (the kernels of the globules,) have a diameter of $\frac{1}{1294}$ of a line; but, on account of the feebleness with which they intercept the light, they are seen with more difficulty than some bodies of a smaller size. Contrary to his former opinion, the author is now convinced from observation that the capillary vessels have membranous parietes.

2. *Thymus Gland.* The author opposes the notion that the thymus gland is not found after twelve years of age. He has found it in almost all individuals between twenty and thirty years; and very often larger than in young children; and he has seen it of considerable size between the ages of thirty and fifty, and has also met with the brownish red remnants of it still later in life. In younger men its form is generally cleft into two parts, as in its original condition: these are generally adherent in the middle only by cellular tissue, so that their decrease appears to commence at this part. The lower cornua never, as in children, descend to the upper part of the pericardium, but frequently extend far into the neck.

The following is the measurement of the thymus gland in some very healthy and well made individuals who had committed suicide:

| Age and Sex. | Length. | Breadth. | Thickness. | Weight. Grains. | Volume. Cubic Inches. | Specific grav. |
|---|---|---|---|---|---|---|
| 25, m. | 34 lines. | 18-25 lines. | 4 lines. | 292.5 | 0.977 | 1.0352 |
| 25, m. | 42 — | 32 — | 2-3 — | 380.3 | 1.156 | 1.0311 |
| 20, m. | | | | 356.5 | 1.083 | 1.0309 |
| 28, f. | 22 — | 16 — | 2 — | 69.2 | 0.211 | 1.0267 |

3. *Intestinal Glands.* Dr. Krause has carefully repeated the observations of Böhm on the intestinal glands (recorded in No. II. of this Journal,) and is quite satisfied of their accuracy. He adds some farther investigations, chiefly respecting the size of the various follicles and crypts of the intestinal canal. He does not, however, regard the glands of Peyer as essentially different from the solitary glands in the jejunum. The manifest differences are, that the glands of Peyer are crowded together and are naked and prominent on the free surface of the mucous membrane, whilst the solitary glands are abundantly beset with follicles. Both are of the same size, between $\frac{1}{8}$ and $\frac{4}{5}$ of a line, their cavity is rather more than half the size of their external circumference, the parietes proportionally thick; the contents of both are not readily squeezed out, but its appearance is that of opaque mucus, the granules of which, flattened and irregularly rounded, are from $\frac{1}{330}$ to $\frac{1}{450}$ of a line in diameter; a size which corresponds with that of the granules of the mucus of many mucous membranes, though they are occasionally found of a larger size. But both the solitary glands and those of Peyer present a character essentially different from that of the glands of other mucous membranes, i. e. a slight roughness of the internal surface of their cavities, produced by a slight prominence of secreting cells, and a plurality of openings, whilst the glands of mucous membranes generally possess but one opening connected with their cavity. It is very rare to find but one opening to either the solitary glands or those of Peyer, the average number of their apertures being from five to ten. The openings traverse the parietes of the follicles in an oblique direction, and it is consequently difficult to assure one's self that they communicate with their cavities. The evidence of this, however, may be obtained thus: Take a large follicle of a gland of Peyer, and open its cavity by removing that half of it which is inserted into the submucous cellular tissue of the intestine; into the little rounded hollow, which is thus rendered visible, insert a very small drop of carmine in solution. On examining the opposite part of the follicle, i. e. that which projects into the intestinal canal where the openings are situated, the red fluid will be seen to escape from these apertures, before the entire follicle becomes coloured by imbibition.—*Archiv. für Anat. Physiol. und Wissent. Medicin.* Von MÜLLER. Heft i., 1837.

---

*On the Relative Motions of the Globules of the Blood and Lymph in the Blood-Vessels of Frogs.* By T. M. ASCHERSON.

IN our Eighth Number we noticed Professor Weber's views on the visible motion of the globules of lymph in the lymphatics of tadpoles, and to it (p. 500) we beg to refer our readers for an account of them. M. Ascherson has been led by his own microscopical observations on the mesentery of the Bufo cinereus, the Rana temporaria, and R. esculenta, and also on the lungs of the two last species, to doubt the correctness of Professor Weber's conclusion that the veins in tadpoles either lie in the cavity of a single large lymphatic, or are surrounded by a plexus of smaller lymphatic vessels; a view which the professor adopted, as that which best explains the appearance, as seen by the microscope, of a transparent border to the veins, into which the globules of the blood are not observed to pass, and which border Professor Weber supposed to be separated by an invisible partition from the venous trunks. According to M. Ascherson, the globules of the blood and those of the lymph move in the cavity of the same vessel; the lymphatic globules, he says, certainly move slower than those of the blood, but he is inclined to believe that the velocity of the one stands in a definite relation to the velocity of the other; the faster the motion of the red globules, the faster also will be the motion of those of the lymph. The latter, however, are apt to lag behind; they differ in shape from, and their surface is rougher than that of the globules of the blood, and hence they do not glide so smoothly along, and their sharp angles, by rubbing against the walls of the vessels, sensibly impede their motion, or sometimes even stop them altogether. A lymphatic globule may thus get attached to the walls of the vein and remain adhering till the projecting edge of a globule of the blood, or the coming up of another lymphatic globule pushes it off and sets it again in motion. In cases where the circulation is proceeding very slowly, the lymphatic globules remain altogether at rest, except when pushed on for a short space by the projecting edge of the globules of the blood. In the centre of the stream of blood the lymphatic globules move evidently quicker than at the edges. The preceding remarks refer more particularly to the appearances in those veins which have a transparent border; but M. Ascherson has convinced himself that there is likewise a difference in the motion of the two varieties of globules in those veins in which the stream of blood appears to be in immediate contact with the walls of the vessel: in them also the lymphatic globules evidently move slower and often get attached to the sides of the vessel.

In the course of his investigations, M. Ascherson was led to pay attention to the elasticity of the globules of the blood, a property which has been denied them by several able physiologists. The globules may be observed in the fine capillaries of the lungs of the frog to assume almost every variety of form and shape in passing through these short and narrow ducts, but this phenomenon becomes extremely apparent when the excised lung is compressed between two plates of glass. From the facility with which they regain their original form M. Ascherson infers that, excepting the elastic fluids, no body is endowed with so much elasticity as the globules of the blood.

M. Ascherson cannot satisfactorily explain the appearance of the transparent border, but he is satisfied that it does not depend upon a separate lymphatic vessel, as he has occasionally, though very seldom, observed a red globule to penetrate the transparent layer. The difference of velocity of the two varieties of globules he explains by the difference of shape, the highly lubricated state of the red, and the rough and gritty surface of the lymphatic globules.

*Müller's Archiv. Jahrgang,* 1837. Heft iv.

---

### *Absence of the Right Lung.—(Cyanosis.)*

The following deformities were found in a child, six weeks after its birth. From birth it had been affected with cyanosis: death was attended with symptoms of congestion in the head. The right lung was entirely wanting: there was only a rudiment of the right bronchus; no right pulmonary artery or vein. The septum ventriculorum cordis was imperfect. The origin of the aorta communicated with both ventricles. The foramen ovale and the ductus arteriosus were both open, the latter widely so, this supplied the left (the only) lung with blood, as the pulmonary artery was imperforate at the base of the heart.

*Wochenschrift für die gesammte Heilkunde.* No. 33. 1837.

